# Host cell stress response as a predictor of COVID-19 infectivity and disease progression

**DOI:** 10.3389/fmolb.2022.938099

**Published:** 2022-08-11

**Authors:** Celine Caillet, Melissa Louise Stofberg, Victor Muleya, Addmore Shonhai, Tawanda Zininga

**Affiliations:** ^1^ Department of Biochemistry, Stellenbosch University, Stellenbosch, South Africa; ^2^ Department of Biochemistry, Midlands State University, Gweru, Zimbabwe; ^3^ Department of Biochemistry and Microbiology, University of Venda, Thohoyandou, South Africa

**Keywords:** SARS–CoV–2, COVID-19, cell stress responses, heat shock proteins, stress proteins, drug target

## Abstract

The coronavirus disease (COVID-19) caused by a coronavirus identified in December 2019 has caused a global pandemic. COVID-19 was declared a pandemic in March 2020 and has led to more than 6.3 million deaths. The pandemic has disrupted world travel, economies, and lifestyles worldwide. Although vaccination has been an effective tool to reduce the severity and spread of the disease there is a need for more concerted approaches to fighting the disease. COVID-19 is characterised as a severe acute respiratory syndrome . The severity of the disease is associated with a battery of comorbidities such as cardiovascular diseases, cancer, chronic lung disease, and renal disease. These underlying diseases are associated with general cellular stress. Thus, COVID-19 exacerbates outcomes of the underlying conditions. Consequently, coronavirus infection and the various underlying conditions converge to present a combined strain on the cellular response. While the host response to the stress is primarily intended to be of benefit, the outcomes are occasionally unpredictable because the cellular stress response is a function of complex factors. This review discusses the role of the host stress response as a convergent point for COVID-19 and several non-communicable diseases. We further discuss the merits of targeting the host stress response to manage the clinical outcomes of COVID-19.

## 1 Introduction

In December 2019, a novel Severe Acute Respiratory Syndrome Corona Virus 2 (SARS-CoV2) was found to be the cause of the Coronavirus disease (COVID-19) outbreak. SARS-CoV2 spread rapidly worldwide, resulting in a pandemic that started in March of 2020 (World Health Organization, 2020). To date, it has infected over 556 million people and caused more than 6.3 million deaths globally (https://www.worldometers.info/coronavirus/). The pandemic has also negatively impacted international travel, trade, education and social interactions across the globe. Coronaviruses (CoVs) have caused three 21st century outbreaks of SARS-related diseases in humans, namely: SARS-CoV of 2004, Middle East Respiratory Syndrome (MERS-CoV) of 2012 and the current SARS-CoV2 of 2019 (Zhang T. et al., 2020; Zhu et al., 2020). SARS-CoV2 is part of the β-coronavirus genus which shares 79% sequence identity with SARS-CoV and 50% with MERS-CoV (Wang Y. et al., 2020). The clinical features of COVID-19 are flu-like symptoms, including nasal congestion, sore throat and dry cough, which can lead to severe pneumonia (Zhu et al., 2020). Severe cases of COVID-19 infection have been reported to be associated with comorbidities such as chronic respiratory and cardiovascular diseases, diabetes, hypertension, and cancer. In these cases, the main causes of death are shock, respiratory and multiple organ failures. This review will mainly focus on the host stress response process to SARS-CoV2 infection and discuss the roles of host cell stress pathways in regulating the progression of the various comorbidities associated with the infection. We further discuss the therapeutic potential of targeting these processes.

## 2 SARS-CoV2 as a cellular stressor

**FIGURE 1:**
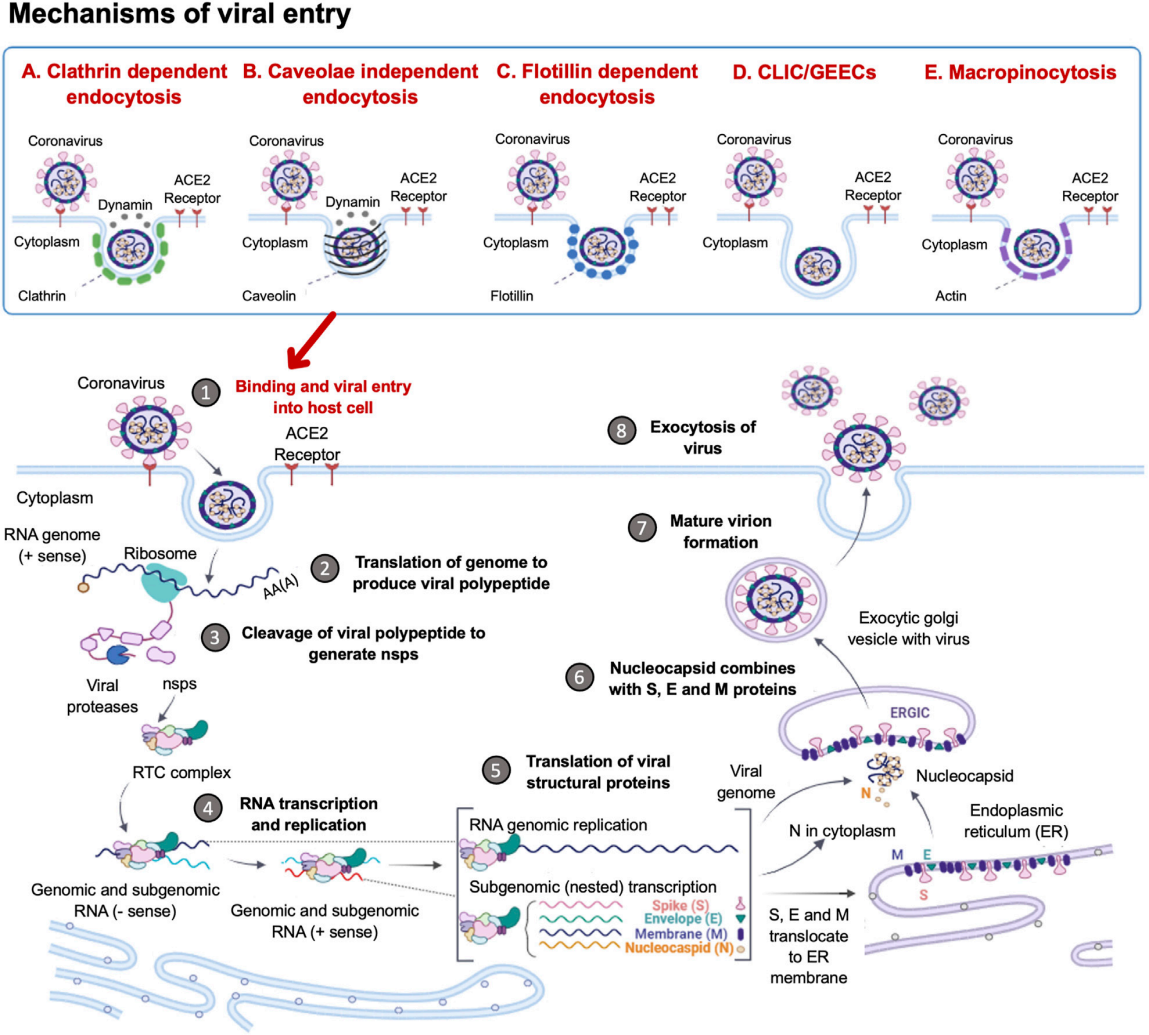
Coronavirus Life Cycle in the Host Cell. The potential mechanisms of viral entry into the host cell include canonical Clathrin-dependent endocytosis and non-canonical pathways such as: Caveolae-independent endocytosis, Flotillin-dependent endocytosis, CLIC/GEEC endocytosis and Macropinocytosis. After viral entry, the replication of the coronavirus in the target cell is initiated. The RNA genome is uncoated, which allows for the initiation of translation using host ribosomes to produce viral polypeptides. These polyproteins are cleaved by proteases to produce non-structural proteins (NSPs), which are responsible for the formation of the replication-transcription complex (RTC). The RTC facilitates the production of genomic and sub-genomic RNA (-sense and + sense) copies. Following the sub-genomic (nested) transcription, viral structural proteins are produced: spike (S), small envelope (E), membrane (M) and nucleocapsid (N) proteins. The ER facilitates the translation of these viral structural proteins and subsequent embedding on the ER membrane. The nucleocapsids assemble in the cytoplasm and bud off to the Endoplasmic Reticulum-Golgi Intermediate Compartment (ERGIC), where they combine with the structural proteins. The accumulation of viral material causes swelling of the Golgi-apparatus, which results in the formation of smooth structures of virions budding off as enveloped smooth vesicles containing the newly acquired envelopes. These mature virions are released through exocytosis. (Figure created using https://biorender.com/).

SARS-CoV2 entry into host cells is an important step in viral infectivity and pathogenesis. To facilitate entry, the viral surface-exposed spike glycoprotein (S) attaches to the host cell receptors. The S protein has a receptor-binding domain (RBD), which contains a cleavage site where it is first preactivated with a proprotein convertase furin. Once it is processed, the RBD binds to the human angiotensin-converting enzyme 2 (ACE2) receptor on the host cells (Zhang H. et al., 2020). Studies have shown that SARS-CoV2 utilises the same ACE2 receptor as SARS-CoV to enter the host cell via its S protein (Hoffmann et al., 2020). The RBD is generally believed to facilitate receptor-mediated endocytosis for viral entry into the host cells (Petersen et al., 2020). However, several other mechanisms of viral entry have been proposed (Figure 1). These mechanisms include: A. The canonical clathrin-mediated endocytosis pathway, which is thought to be ACE2 receptor-dependent and pH-sensitive (Milewska et al., 2018; Yang and Shen, 2020); B. The non-canonical caveolae independent endocytosis pathway (Szczepanski et al., 2018), which involves anchored lipid rafts and micropinocytosis (Glebov, 2020); C. Flotillin-1-associated endocytosis; D. Clathrin-independent carrier (CLIC)/glycosylphosphatidylinositol-anchored protein-enriched endosomal compartment (GEEC) endocytosis and E. Macropinocytosis (Glebov, 2020). These various entry pathways are thought to be utilised by the virus depending on the target cell types. It has been reported that more than one mechanism of host entry could be employed by SARS-CoV2 (Yang and Shen, 2020).

Following the entry of the virus into the host cell via an endosome, lysosomal proteases facilitate viral uncoating to release the RNA genome in the cytoplasm. When the released positive-sense RNA genome is translated at the ribosome to produce viral polypeptides and structural proteins. This is followed by the transcription of the viral genes with subsequent packaging in the nucleocapsids (Figure 1). In the first event, the positive-sense RNA genome is translated into two large viral polyproteins, namely: PP1a and PP1b, that are encoded from approximately two-thirds of the viral genome (Chen et al., 2020; Hidalgo et al., 2021). These viral polyproteins are cleaved by viral encoded chymotrypsin-like proteases (3CLpro) and papain-like proteases, to yield 16 non-structural proteins (NSP1-16) (Andersen et al., 2020; Uddin et al., 2020). The second event is facilitated by NSPs that form the replication-transcription complex (RTC) which facilitates the transcription and replication of the remaining one-third of sub-genomic mRNAs encoding for the four main structural proteins, namely: spike (S) receptor binding, envelope (E), membrane (M) and nucleocapsid (N) (Gordon et al., 2020; Kim et al., 2020). Thereafter, the structural proteins are embedded onto the Endoplasmic Reticulum-Golgi Intermediate Compartment (ERGIC) or on the Endoplasmic Reticulum (ER). These newly synthesised genomic RNA molecules and proteins accumulate to form the nucleocapsid. The modified ERGIC containing the viral structural proteins, buds off the ER and encloses the nucleocapsid to form a mature virion (Klumperman et al., 1994). The new virions are prepared for secretion through exocytosis via the Golgi apparatus. During this process, the ER membranes are depleted and the cell protein folding machinery is hijacked to process these viral proteins. These events exert stress upon the infected cell. Interestingly, it has previously been established that the stress-induced expression of heat shock proteins (Hsps) in *Drosophila* restricts viral infectivity (Merkling et al., 2015).

## 3 Overview of stress proteins

**FIGURE 2:**
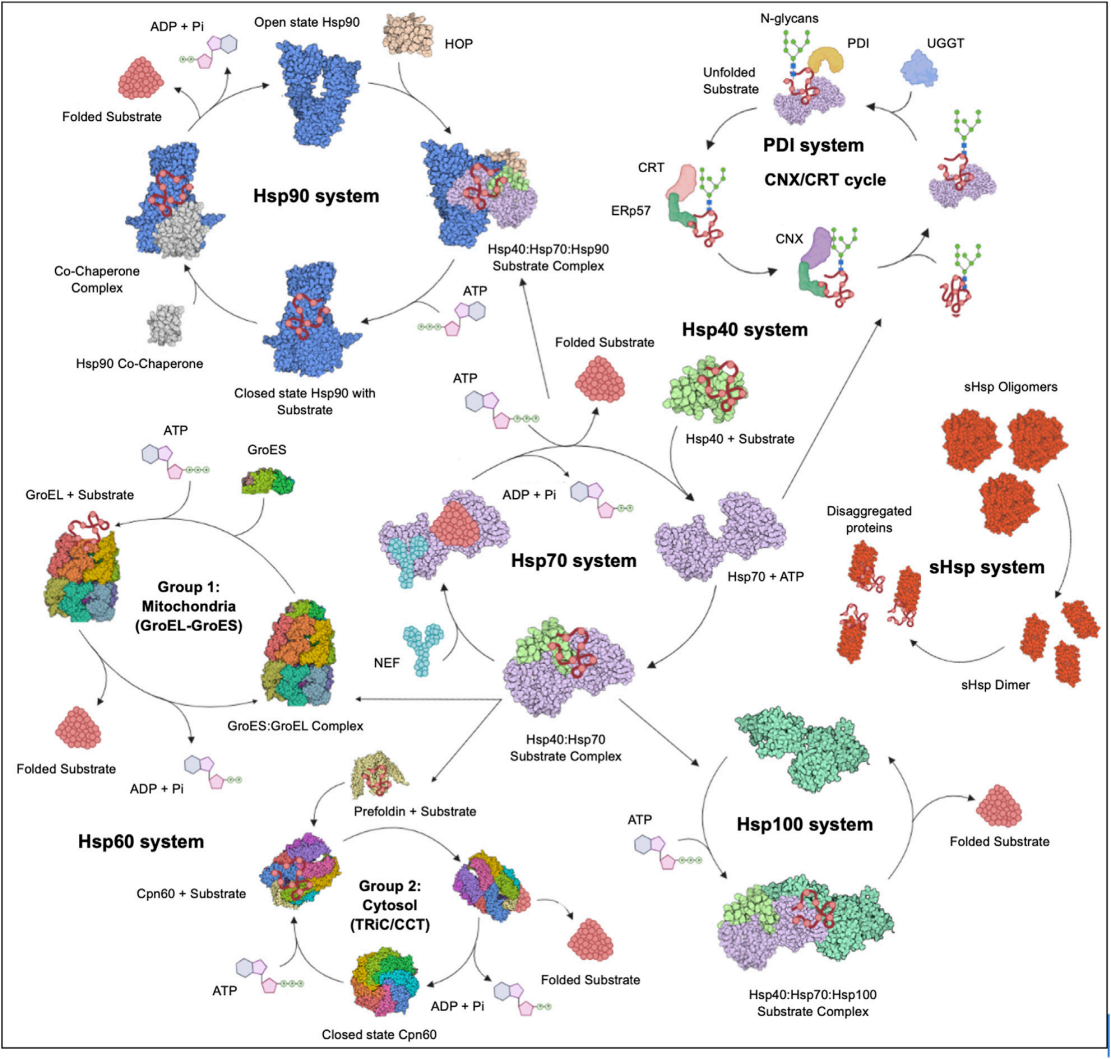
The Hsp chaperone system. The Hsp40 chaperone system recruits nascent substrate proteins and transports them to the Hsp70 folding system. Thereafter, the folded clients are transferred over to the Hsp90 system for activation or assistance to form multiple protein complexes. The more complex substrates are brought to the Hsp60 (GroEL) and TRiC systems. Unfolded proteins are transferred to the Hsp100 system for disaggregation in cooperation with the sHsp system. The CRT and CNX recruit Hsp folded glycoproteins to PDIs for further folding and disulphide bond stabilisation. Figure adapted from the HSP information resource database (http://pdslab.biochem.iisc.ernet.in/hspir/index.php).

Stress proteins (SPs) are a set of molecular chaperones, whose expression is upregulated in response to cellular stress. These SPs are involved in cytoprotection by facilitating protein folding and unfolding, protein activation and the assembly of protein complexes. Cellular stress response modulates pathways that stimulate cell survival or cell death and the dysregulation thereof. For this reason, cell stress response is implicated in various human diseases including cardiovascular diseases, neurodegenerative diseases, cancer and some other infectious diseases. Hsps, along with the predominantly ER localised Protein Disulphide Isomerases (PDI) (Wan et al., 2020), constitute key components of the cellular stress response machinery. Hsps are generally classified into several different families based on their average molecular sizes in kDa as well as sequence conservation. They fall within the following key groupings: Hsp110, Hsp100, Hsp90, Hsp70, Hsp60, Hsp40 (also known as J domain proteins; JDP) and lastly, small Hsps (sHsps) (Figure 2). Due to their central role, Hsps are implicated in several cellular pathways and are inherently linked to various pathologies (Favatier et al., 1997; Edkins et al., 2018).

### 3.1 The Hsp100 protein family

In humans, there are six Hsp100 members with diverse functions. Two members exhibit protein disaggregation capabilities, while the other four members are proteases (represented by caseinolytic protease (Clp)). Structurally, Hsp100s are grouped into two groups, type 1 and type 2. Type 1 refers to members with two AAA+ ATPases domains, namely the NBD 1 and NBD2. These domains are characterised by the presence of two walker motifs (1 and 2) and a middle domain between the NBDs (Zolkiewski et al., 2012). Hsp104/ClpB, ClpA, ClpC and Hsp78 constitute the type I cluster. The type 2members of the Hsp100 members, ClpP and Hsv, possess a single NBD2 but lack the middle domain. Hsp100 proteins have been implicated in neurodegenerative diseases and other protein-folding-related diseases. Their implication in these pathologies is based on their role in suppressing and reversing protein aggregation (Ferrari et al., 2018). As a disaggregase, Hsp100 occurs in complex with Hsp70, Hsp110 and Hsp40 (Kaimal et al., 2017; Lin et al., 2022). This highlights that while the functions of the various Hsps are unique, they also cooperate to manage cellular stress.

### 3.2 The Hsp90 protein family

Five human Hsp90s are localised within the cytosol, ER and mitochondria. The three cytosolic paralogs include: the stress-inducible α-Hsp90 (HSP90AA2/HSPC2), the truncated chimeric Hsp90 (HSP90AAA1/HSPC1) and the housekeeping β-isoform (HSPB1/HSPC3) (Chen et al., 2005). The ER and mitochondria host the 94 kDa glucose-regulated protein (Grp94/HSPC4) and the tumour necrosis factor receptor-associated protein-1 (TRAP 1/HSPC5) respectively (Kampinga et al., 2009). Structurally, these proteins share a conserved domain architecture that comprises the N-terminal ATPase domain, the middle domain with substrate binding capability and the C-terminal dimerization domain (Jackson, 2012). Hsp90s are ATP-dependent molecular chaperones that play a central role in protein homeostasis (Obermann et al., 1998; Chakraborty and Edkins, 2021). The function of Hsp90 is regulated by several co-chaperones (Bachman et al., 2018). In the ADP-bound state, the clients/substrates are recruited into an early complex consisting of the Hsp70/Hsp40/Hsp-interacting protein (HIP) and the Hsp90/Hsp70-organising protein (Hop) (Luengo et al., 2019). ATP hydrolysis is activated by the Hsp90 ATPase activator 1 (AHA1; Oroz et al., 2019). Following nucleotide exchange, the Hsp90 forms a mature complex with co-chaperones p23, p50, cell division cycle 37 (cdc37) and immunophilins (Biebl et al., 2020). Furthermore, post-translational modification of Hsp90 through phosphorylation (Xu et al., 2019) and acetylation (Mollapour and Neckers, 2012), regulates its functional specificity (Prodromou, 2016). Host Hsp90 substrates that are associated with the possible uptake of viruses include those responsible for transcription, translation, mitochondrial function, kinetochore assembly, centrosome function and maintenance of the cell cycle (Lubkowska et al., 2021). Hsp90 also facilitates membrane trafficking and membrane deformability during the release of exosomes (Lauwers et al., 2018). There is a wide array of Hsp90 clients, most of which are molecules involved in signal transduction. Hsp90 facilitates the conformational maturation of several of its client. In addition, Hsp90 is also involved in the ordered assembly and stabilisation of subunits of multiprotein complexes (Makhnevych and Houry, 2012). This highlights a possible central role of this chaperone in viral replication within the host. To this end, inhibitors of Hsp90 are of interest as possible therapies against COVID-19 (Ramos and Ayinde, 2021; Wyler et al., 2021; Biancatelli et al., 2022).

### 3.3 The Hsp70 family

At least seventeen members of the Hsp70 family of chaperones are found in humans. Hsp70s are grouped into two subfamilies: canonical members (Hsp70) and non-canonical members (Hsp110) subgroup. There are 13 canonical Hsp70s which resemble the prokaryotic Hsp70, represented by *E. coli* DnaK. They include the cytosolic Hsp70-1/Hsp72/HSPA1A, HSPA1B/Hsp70-2, HSPA1L/Hum70t, HSPA2, Hsp70B/HSPA6, HspA7/Hsp70-7, Hcs70/HSPA8, HSPA12A/FLJ13874, HSP112B/RP23-32L15.1, HSPA13/Stch and HspA14/Hsp70-4; the ER localised Grp78/Bip/HSPA5 and the mitochondrial localised mortalin/GRP75/HSPA9. Hsp110 members include, the cytosolic Hsp105/HSPH1, Apg-2/Hsp110/HSPH2, Apg-1/HSPH3 and the ER localised Grp170/HSPH4 (Easton et al., 2000; Kampinga et al., 2009; Chakafana and Shonhai, 2021). Structurally, Hsp70s are composed of an N-terminal nucleotide-binding domain (NBD) that exhibits ATPase activity and a C- terminal substrate-binding domain (SBD). The Hsp110 subfamily displays a similar domain architecture, however, they have a long acidic insertion in the SBD, making them larger members (Oh et al., 1999; Chakafana et al., 2019). Hsp110 functions as a chaperone, whilst also facilitating nucleotide exchange for its canonical Hsp70 counterparts (Dragovic et al., 2006; Andreasson et al., 2008). The Hsp70 chaperone plays a central role in the folding of nascent polypeptides released from the ribosomes. It also refolds misfolded proteins and as well as facilitates the assembly of multiprotein complexes (Figure 2). In addition, Hsp70 also cooperates with Hsp90 and Hsp60 to facilitate the maturation of some of its clients (Luengo et al., 2019; Wang et al., 2022).

The primary co-chaperone of Hsp70s are the Hsp40 proteins (JDP). Hsp40s are composed of a conserved J domain that facilitates the interaction with Hsp70, activating ATP hydrolysis by the latter (Cintron and Toft, 2006; Kampinga et al., 2009). Hsp40s are also known to recruit substrates to Hsp70 and are therefore called substrate scanners (Fan et al., 2003). Hsp70 has a high affinity for substrates in the ADP-bound state. To facilitate substrate release, the nucleotide exchange factors (NEFs), such as Hsp110, exchange ADP for ATP (Dragovic et al., 2006; Alderson et al., 2016). These functions contrast with HIP, which locks Hsp70 in the ADP-bound state (Nollen et al., 2001). Therefore, the action of NEFs and HIP determines the substrate residency time on Hsp70, which influences substrate fate. Hsp70 was found to be one of the distinct biomarkers circulating in COVID-19 ICU cases (Fraser et al., 2020). Considering the cytoprotective role of Hsp70, it has been proposed that the periodic fever conditions associated with COVID-19 infections, may benefit the host by stimulating the expression of this chaperone (Guihur et al., 2020).

**FIGURE 3:**
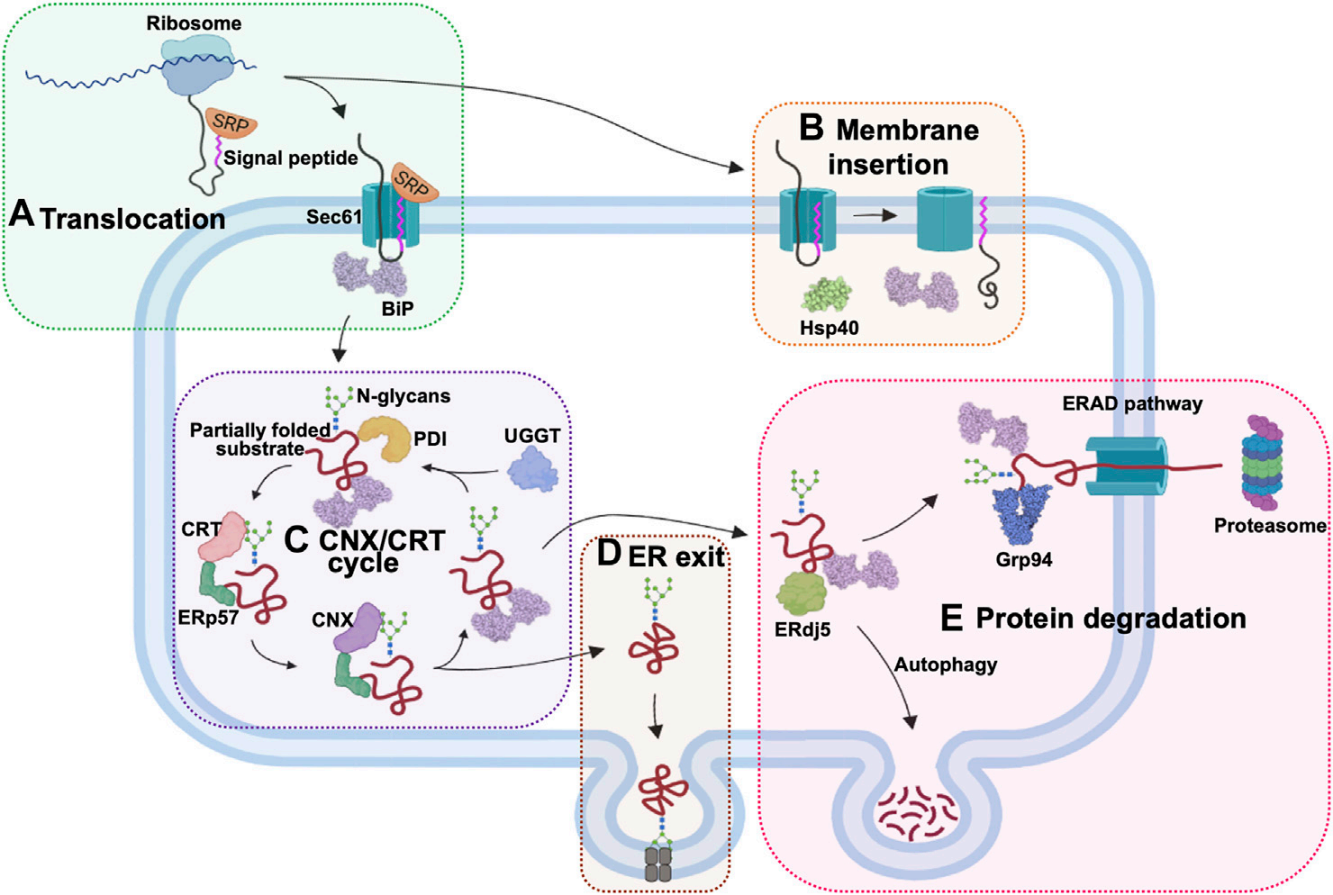
The role of ER chaperones in proteostasis. The ER-localised chaperones have numerous roles in this cellular organelle. **(A)** ER chaperones enable the translocation of proteins into the ER. Newly synthesized proteins are targeted by the signal recognition particle (SRP) as they emerge from the ribosomes. The SRP-bound protein enters the ER through the Sec61 translocon protein channel and is bound by Hsp70 (Grp78/BiP) inside the ER lumen for active import. **(B)** Chaperones also facilitate post-translational ER membrane insertion of proteins. **(C)** The CNX/CRT chaperone system facilitates proper glycosylation of proteins targeted for various cellular compartments. **(D)** ER chaperones are involved in processing the exit of the proteins via the ERGIC pathway. **(E)** Proteins that are not properly folded are bound by ERdj5, BiP, and Grp94 in the ER and channelled for degradation by autophagy or through the ERAD pathway in the cytosol.

The ER-based chaperones have widespread functions in nearly every stage of protein processing (Figure 3). During protein import into the ER lumen, newly synthesised polypeptides that emerge from the ribosomes, are recognized by the signal recognition particle (SRP), which transports these proteins to the ER membrane for translocation via the Sec61 channel (Haßdenteufel et al., 2018; Jomaa et al., 2022). The ER-resident Hsp70 (Grp78/BiP), binds incoming peptides and actively threads them into the ER lumen (Craig, 2018; Haßdenteufel et al., 2018). BiP and the ER-resident Hsp40 (ERdj5), play a role in the post-translational insertion of proteins into the ER membrane (Araki and Nagata, 2012). They also facilitate the processing of aggregated membrane proteins, by earmarking them for degradation. Additionally, ER chaperones also facilitate the export of proteins from the ER through their involvement in the ERGIC pathway (Ito and Nagata, 2019). Irreparably misfolded proteins are destroyed by autophagy or redirected to the ER-associated degradation (ERAD) pathway for destruction in the proteasomes located in the cytosol (Oikonomou and Hendershot, 2020; Braakman and Hebert, 2021). This pathway involves the ER-resident chaperones such as ERdj5, BiP and Grp94, which bind and target substrates for degradation (Adams et al., 2019).

### 3.4 The Hsp60 chaperonins

Hsp60 proteins are ATP-dependent chaperonins and are classified into two main groups, namely, type 1 and type 2 (Okamoto et al., 2017; Ishida et al., 2018). Type 1 chaperonins are mainly found in the mitochondria of eukaryotes and in the cytoplasm of prokaryotes (GroEL in *E. coli*). This class of chaperonins form a 7-member ring back-to-back complex with a central core, which then requires Hsp10 (GroES in *E. coli*) to close the core, functioning as a lid (Enriquez et al., 2017). The type 2 chaperonins, which include the cytosolic TCP1-ring complex (TRiC), are not well studied but are found in the archaeal chromosome and eukaryotic cytosol. They form a similar dimerization of the 8-9 protomer complexes to make 16–18 subunits joined end to end, forming a barrel structure with a central core (Ishida et al., 2018). Similar to type 1 chaperonins, the central core is closed by the Hsp10 protein. Some Hsp60s escape the mitochondria and translocate to the circulatory system, where they are known to induce proinflammatory cytokines. For this reason, Hsp60 is implicated in hypertension and is therefore thought to aggravate COVID-19 related complications (Jakovac, 2020).

### 3.5 Small heat shock proteins

In humans, there are eleven members of the small heat shock protein (sHsp) family of chaperones. They include HSPB1/Hsp25, HSPB2/Hsp27, HSPB3/Hspl27, HSPB4/crystallin α-A, HSPB5/crystallin α-B, HSPB6/Hsp20, HSPB7/cvHsp, HSPB8/Hsp22, HSPB9/FLJ27437, HSPB10/ODF1 and HSPB11/Hsp16.2 (Kampinga et al., 2009). Structurally, sHsps have a conserved α-crystallin domain, sandwiched with a variable N-terminal region and the C-terminal regions. These sHsps exhibit holdase chaperone activity which is ATP-independent. They function in complexes to minimise protein unfolding and serve as holdases (Haslbeck et al., 2019). The most studied sHsps are the Hsp27, crystallin α-A and crystallin α-B. Of these, only Hsp27 has been implicated in COVID-19 pathology (Wendt et al., 2021).

### 3.6 Protein disulphide isomerases

Protein Disulphide Isomerases (PDIs) are oxidoreductases that catalyse the enzymatic reduction and isomerization of disulphide bridge formation (Appenzeller-Herzog and Ellgaard, 2008). In eukaryotes, almost a third of the cellular proteome contains disulphide bonds (Mahmood et al., 2021). In the ER, the PDIs stabilise and promote the folding of client proteins into three-dimensional structures (Kranz et al., 2017). In humans, there are 19 PDIs localised in the ER (Ellgaard and Ruddock, 2005). Generally, the PDIs are characterised by the CXXC active site motif, where the cysteine residues take part in the exchange of disulphide bond formation to stabilise client proteins. PDIs are also involved in ER protein degradation (ERAD) and calcium level regulation (Kramer et al., 2001; Riemer et al., 2011). The most studied PDI is PDIA3 (ERp57/Grp58), which is comprised of the canonical four thioredoxin domain structure of the **a-b-b-a** domain organisation. In general, the **a**-domains of PDIs contain the catalytic CXXC active site motif, which can exhibit thiol-sulphate reductase, oxidase or isomerase activity (Darby and Creighton, 1995; Chichiarelli et al., 2022). The **b**-domains bind substrates with high affinity to facilitate isomerization (Klappa et al., 1998). The PDIA3 **b**-domains do not directly interact with substrate proteins but rather indirectly through their association with lectins, calreticulin (CRT) and calnexin (CNX) (Oliver et al., 1999; Molinari et al., 2004). The CRT and CNX recruit glycoproteins to PDIA3 for correct folding and disulphide bond stabilisation. If the PDI fails to achieve a competent fold, the substrate protein undergoes re-glycosylation by the glucose:glycoprotein:glucosyl transferase (UGGT) to repeat the cycle (Kozlov and Gehring, 2020; Mahmood et al., 2021). Therefore, PDIs play an essential role in protein disulphide bond formation and protein glycosylation quality control mechanisms that are thought to be essential for SARS-CoV2 protein maturation (Fu et al., 2020). Abnormalities in these protein quality control systems in all cell organelles, have severe consequences for the cell and have been implicated in several diseases (Parakh and Atkin, 2015; Chamberlain and Anathy, 2020). Protein folding aberrances are at the centre of diseases such as cancer, neurodegenerative disorders, metabolic diseases, and infections (Gámez et al., 2018).

## 4 COVID-19 susceptibility profiles

SARS-CoV2 pathogenesis is mainly exacerbated by underlying cellular stress, which is more pronounced in patients with comorbidities such as diabetes, cardiovascular disease, hypertension and obesity among others (Sanyaolu et al., 2020). Generally, viral infections are associated with inflammation, a hallmark of COVID-19 pathology (Varga et al., 2020), which further puts strain on the protein folding system (Kuppalli and Rasmussen, 2020; McGonagle et al., 2020). The role of Hsps in immunomodulation is well established and appears to be a function of their circulating levels (Zininga et al., 2018). Thus, the expression profiles of these proteins in various disease conditions could serve as biomarkers of disease severity and patient outcomes (Table 1).

**TABLE 1:** The role of Hsp expression levels in diseases.

**Disease (COVID-19 co-morbidity state)**	**Implicated Hsp**	**Implication on inflammation/disease severity**	**References**
**Chronic lung diseases**			
•Asthma	Hsp70↑	Triggers both pro- and anti- inflammatory responses	Choi et al. (2021)
			Shevchenko et al. (2020)
•Chronic obstructive pulmonary diseases (COPD)	Hsp27↑; Hsp70↑	Elevated serum concentrations of Hsp27 and Hsp70 is a strong predictor of mortality	Gerayeli et al. (2021)
			Celli and Wedzicha, (2019)
			Zimmermann et al. (2020)
			Dong et al. (2013)
•Interstitial lung diseases (ILD)	Hsp70↓; Hsp90↑	Reduced Hsp70 and increased Hsp90 trigger pro-inflammatory responses	Skolnik and Ryerson, (2016)
			Cottin, (2013)
			Sellares et al. (2019)
			Chen et al. (2018)
			Storkanova et al. (2018)
**Chronic kidney diseases**	Hsp27↑; Hsp72↑ Hsp90↑	Reduced Hsp27 and Hs72 triggers pro-inflammatory responses and protects cells	Marzec et al. (2009)
		Increased Hsp90 enhances oxidative stress and inflammation	Musial et al. (2010)
			Musial and Zwolińska, (2011)
**Obesity**	Hsp60↑; Hsp72↑; Hsp90↑; Hsp70↑; Grp94↑; Hsp40↓	Increased expression of Hsps is linked to increased inflammation	Sell et al. (2017)
		Decreased Hsp40 may be implicated in regulation of insulin resistance	Märker et al. (2012)
			Tiss et al. (2014)
**Diabetes**	Hsp60↓; Hsp70↓; Hsp72↓; Hsp90↑	Reduced expression of Hsp 60 and Hsp70 is associated with increased inflammation	Bijur et al. (2000)
			Archer et al. (2018)
			Khadir et al. (2018)
		Decreased Hsp72 expression is linked to insulin resistance	Lee et al. (2013)
			Zilaee and Shirali, 2016
		Upregulated Hsp90 contributes to inflammation and vascular complications	Amawi et al. (2019)
**Cardiovascular diseases**	Hsp27↓; Hsp60↑; Hsp90↑; Hsp70↑	Low Hsp27 associated with cardiac disease and death	Jaroszyński et al. (2018)
			Duan et al. (2020)
		Increased Hsp60, Hsp70 and Hsp90 associated with atherosclerosis and cardiac failure	Ranek Mark et al. (2018)
			Rodriguez-Iturbe and Johnson, (2018)
**Cancers**		Increased levels of Hsps in cancer cells lead to cancer cell proliferation, metastasis, immunomodulation, and prevention of apoptosis	
•Neuroblastoma	Hsp27↓; Hsp60↑		Wu et al. (2017a)
•Renal	Hsp70↓		
•Pancreatic	Hsp90↑; Hsp110↑		
•Ovarian	Hsp40↑; Hsp90↑		
•Breast	Hsp27↑; Hsp40↑; Hsp60↑; Hsp70↑; Hsp90↑; Hsp110↑		
•Colon	Hsp40↑; Hsp70↑		
•Lung	Hsp27↑; Hsp40↑; Hsp60↓; Hsp70↑; Hsp90↑		Luo et al. (2020)
			Mittal and Rajala, (2020)
•Liver	Hsp27↑; Hsp60↑; Hsp70↑; Hsp90↑; Hsp110↑		Cedrés et al. (2018)
			Sun et al. (2018)
			Jiang and Shen, (2020)
			Wu et al. (2017a)

The level of expression of Hsps present in each disease state is indicated with the arrow direction for upregulated (↑) and downregulated (↓) expression levels.

### 4.1 Chronic lung diseases

Chronic lung diseases include a wide array of diseases such as asthma, Chronic Obstructive Pulmonary Diseases (COPD), Interstitial Lung Diseases (ILD), cystic fibrosis, lung cancer and chronic pneumonia to name a few (Cottin, 2013; Celli and Wedzicha, 2019). Chronic lung diseases cause excessive inflammation, immune dysregulation, and impaired repair processes, which ultimately leads to tissue damage and diminished organ function (Meikle et al., 2021). Chronic inflammation is a prominent symptom of chronic lung diseases and causes elevated levels of reactive oxygen species (ROS) in cells, resulting in oxidative stress (Hulgan et al., 2003; Sharif-Askari et al., 2021). Excessive ROS may stimulate prolonged inflammatory responses and signalling cascades that damage cells, which may lead to apoptosis (Chatterjee, 2016; Ivanov et al., 2017).

Hsp70 is variably expressed depending on the nature of the conditions affecting the lungs. The elevated expression of Hsp70 associated with asthma may trigger either pro- or anti-inflammatory pathways, due to its diverse immunomodulating effects (Shevchenko et al., 2020). Hsp70 and Hsp27 expression levels were elevated in lung tissues of patients with COPD, when compared to healthy controls, and were correlated to disease severity (Dong et al., 2013; Zimmermann et al., 2020). In Idiopathic Pulmonary Fibrosis (IPF), one of the common interstitial lung diseases, Hsp70s were observed to be downregulated in response to an increase in the profibrotic molecules, IGFBP5 (insulin-like growth factor-binding protein 5) or (TGF β1) transforming growth factor-β1 (Sellares et al., 2019). Thus, Hsp70 suppression perpetuates fibrosis development in the human fibroblasts. Hsps alleviate oxidative stress through their active roles in the refolding of damaged proteins and PDIs are important for maintaining a redox balance inside the cells suppressing the development of pulmonary fibrosis (Parakh and Atkin, 2015; Marinova et al., 2020; Tanguy et al., 2021).

One of the major complications of COVID-19 is the development of acute respiratory distress syndrome (ARDS), a condition that tremendously impairs the ability of the lungs to absorb oxygen (Meikle et al., 2021). In a 2007 study, lung injury was induced in rats, whereafter the rats were administered with adenoviral vectors expressing Hsp70 proteins (Weiss et al., 2007). It was observed that Hsp70 limited NF-ĸB activation, which in turn limited the proteasomal degradation of IĸBα. Indeed, Hsp70 expression is reportedly elevated in ARDS (Alreshidi et al., 2021), and is known to inhibit intracellular proteasomal degradation (Ryu and Ha, 2020). It was observed that Hsp70 limited NF-ĸB activation, which in turn limited the proteasomal degradation of IĸB kinase signalosome, thereby suppressing inflammation (Weiss et al., 2007). Thus, by suppressing inflammation, Hsp70 expression may indirectly regulate COVID-19 pathology (Sharif-Askari et al., 2021). Additionally, it was reported that several oxidative stress genes are upregulated during Coronavirus infection and expression of these genes is thus likely induced by SARS-CoV2 (Sharif-Askari et al., 2021). Hsp70 being a central player in preventing the accumulation of oxidative stress, might be similarly affected by SARS-CoV2. Although not yet experimentally confirmed, there might be a link between the dysregulation of Hsp70 and other stress proteins and the severity of SARS-CoV2 infection.

### 4.2 Chronic kidney disease

Chronic Kidney Disease (CKD) is a condition characterised by glomerulosclerosis and interstitial fibrosis (Musiał and Zwolińska, 2011). CKD is commonly a result of stress inflicted on hepatocytes from various sources such as, uremic toxins, pro-inflammatory molecules, reactive oxygen species, pro-apoptotic molecules, infectious agents, and dialysis (Nayak Rao, 2016). Several studies reported that the level of Hsp72 expression was upregulated in patients with CKD (Musial and Zwolińska, 2011; Lebherz-Eichinger et al., 2012; Morales-Buenrostro et al., 2014). The presence of uremic toxic may cause increased expression of Hsp72, which has been shown to inhibit the proliferation and apoptosis of renal tubular cells, resulting in reduced renal fibrosis (Pan et al., 2020). Chronic kidney damage is also thought to be associated with noxious conditions where upregulated Hsp72 suppresses apoptosis (Rao, 2016). Although research regarding the role of Hsps in CKD is limited, more work on the role of these proteins in renal dialysis has been conducted. It has been observed that renal ischemia-reperfusion injury (IRI), incurred during renal dialysis, resulted in an induction of Hsp72 (43-fold increase) and Hsp27 (12-fold increase) (Nayak Rao, 2016). Additionally, Hsp70 offers protective properties from renal IRI that include, cytoskeletal stabilization, anti-inflammatory effects, anti-apoptotic properties, and influence over the stimulation of regulatory T-cells (Nayak Rao, 2016). These functions of Hsp70 and sHsp are potentially important in reducing further complications upon the onset of SARS-CoV2 (ERA-EDTA Council et al., 2021). Furthermore, elevated levels of Hsp90 observed in CKD patients are associated with increased oxidative stress and inflammation (Musial and Zwolińska, 2011).

### 4.3 Obesity

Obesity is a metabolic syndrome generally linked to the increased severity of several non-communicable diseases such as, type 2 diabetes mellitus (T2DM), cardiovascular diseases (CVD) and certain cancers (Blüher, 2019). The accumulation of adipose tissue and the increases in energy inputs associated with obesity often triggers chronic inflammation in the fatty tissues (Sell et al., 2017). This inflammation results in an increase in proinflammatory cytokines, both locally and systemically (Lehr et al., 2012; Sell et al., 2012; Tiss et al., 2014). The role of Hsps in inflammation in obese individuals is not well established, as conflicting results have been reported. For example, increased levels of Hsp60, Hsp72, Hsp90, Hsp70 and Grp94 released from adipocytes under stressful conditions have been shown to act as adipokines, linking their expression to obesity and chronic inflammation (Märker et al., 2012; Tiss et al., 2014; Sell et al., 2017). Conversely, individuals with obesity and insulin resistance were reported to exhibit suppressed heat shock response (HSR) activity which by extension results in reduced Hsps expression, as insulin signalling is essential to HSR activity (Di Naso et al., 2015; de Lemos Muller et al., 2018; Bruxel et al., 2019). This contrasts with normal inflammatory conditions where the HSR upregulates Hsp production, which counteracts the inflammatory response (Singh and Hasday, 2013; Zininga et al., 2018; Krause et al., 2020). The decreased HSR activity in obese individuals could be responsible for the dysregulated inflammation and negative prognosis in individuals infected with SARS-CoV-2 (Krause et al., 2020). Unlike the other Hsps, Hsp40 expression is decreased in obese individuals. A study reported that normal levels of Hsp40 were restored upon exercise, suggesting a possible role for this protein in the regulation of insulin resistance and thus mitigating against obesity (Tiss et al., 2014).

### 4.4 Diabetes

The metabolic disorder, T2DM, is characterised by the dysregulation of insulin production and activity, leading to chronically elevated levels of sugar in the blood. The disruption of insulin production associated with diabetes, in turn, disrupts the insulin signalling pathway which is a crucial part of the HSR system. Insulin inhibits the activity of the glycogen synthase kinase-3β (GSK-3), which suppresses the activation of HSF-1, abrogating its interaction with heat shock elements, an important step in regulating transcription of HSP genes (Bijur et al., 2000). Without insulin to inhibit the activity of GSK-3, HSR activity is downregulated and so is the expression of Hsps. Therefore, patients with diabetes are more susceptible to severe infections as they are unable to regulate the resulting inflammation, culminating in further complications (Krause et al., 2020). This partially explains the increased case fatality in individuals infected with COVID-19 when compared to non-diabetic patients (Xue et al., 2020). T2DM patients display dysregulated levels of Hsp60, Hsp70 and Hsp72, which contribute to inflammation and insulin resistance and vascular complications (Khadir et al., 2018; Zilaee and Shirali, 2016; Amawi et al., 2019).

### 4.5 Cardiovascular diseases

Cardiovascular diseases (CVD) are characterised by cellular stress, in which a collection of cardioprotective Hsps are released in the heart (Henderson and Pockley, 2012; Ranek Mark et al., 2018). Several Hsps including Hsp27, Hsp60, Hsp70 and Hsp90, are secreted and released at different rates during coronary stress (Jaroszyński et al., 2018; Duan et al., 2020; Krishnan-Sivadoss et al., 2021). A high secretion of Hsp27 has been shown to offer some cardioprotection, whilst low Hsp27 serum levels, especially in older patients, has been associated with carotid atherosclerosis and oxidative stress. This leads to an increased risk of cardiovascular disease and sudden cardiovascular death (Jaroszyński et al., 2018). Upon cardiac injury, Hsp60 is released into the extracellular fluid, where it activates the body’s innate immunity through the induction of a proinflammatory state in the heart. The subsequent increase in the production of the tumour necrosis factor, TNF-α, facilitates apoptosis and thus attributes to the progression of heart failure (Duan et al., 2020).

Upregulated Hsp60 expression has been found in atherosclerotic lesions and has increased the risk of atherosclerosis (Grundtman et al., 2011). In addition, the cross-reactivity of the immune system with autologous Hsp60 and Hsp70 results in T-cell adhesion to endothelial cells and the initial inflammatory response of atherosclerosis (Rodriguez-Iturbe et al., 2019; Duan et al., 2020). Consequently, autoantibodies produced against either Hsp60 or Hsp70 were reported to exacerbate atherosclerosis (Schett et al., 1997; Stocker and Keaney, 2004; Wick et al., 2014) and hypertension (Rodriguez-Iturbe et al., 2019; Romagnoli et al., 2020). Therefore, Hsp60 and Hsp70 are intricately involved in the development and progression of atherosclerosis and subsequent complications in other diseases. Notably, there are increasing reports linking the induction of Hsp70, Hsp90 and co-chaperones to heart failure (Ranek Mark et al., 2018; Rodriguez-Iturbe and Johnson, 2018).

Patients who become infected with SARS-CoV2, whilst having underlying cardiovascular conditions, have a higher risk of developing a severe infection, myocarditis, and blood clots, which increases the chance of death (Huang et al., 2020; Srivastava, 2020). This is due to a combination of the effects of a viral infection, coupled with the stress caused by the underlying cardiovascular conditions (Srivastava, 2020). Consequently, the intricate involvement of Hsps in several cardiovascular diseases most likely influences the severity of COVID-19 in these patients. Hsp60 appears to be the most studied Hsp implicated in cardiovascular diseases and COVID-19 (Jakovac, 2020). For example, one study hypothesized that the high levels of Hsp60 present in the plasma of hypertensive patients contribute to the cytokine release syndrome (Song et al., 2020). This is the main mechanism responsible for the third hyperinflammatory phase of COVID-19, which often leads to heart failure (Romagnoli et al., 2020). SARS-CoV2 also causes substantial tissue damage, which can result in the release of intracellular Hsp60 into the plasma. Subsequently, this causes an increase in pre-existing Hsp60 levels and could result in systemic hyper inflammation, causing damage to multiple organs (Romagnoli et al., 2020). It was also found that Hsp60 levels in the plasma positively correlate to acute lung injury and systemic inflammatory responses in patients with no prior pulmonary trauma (Pespeni et al., 2005). Although more research is required to fully understand the role of Hsps in heart failure, there is a common consensus that these proteins have important therapeutic and diagnostic considerations in COVID-19.

### 4.6 Cancer

Cancer is a disease state during which abnormal cells grow rapidly and uncontrollably, such that they have harmful effects on tissues and organs. Cancerous cell propagation is highly dependent on stress proteins to assist in the folding of improperly folded and mutated proteins, for their continued dysregulated growth and development (Wu et al., 2017a; reviewed in Chakafana and Shonhai, 2021). The underlying causes and mechanisms involved in Hsp expression are not fully understood. For example, Hsp27 and Hsp70 expression is downregulated in neuroblastoma and renal cancer respectively (Wu P. et al., 2017). Conversely, in hepatocellular carcinoma (HCC), the upregulation of Hsp27 plays a cytoprotective role in preventing cancerous cell apoptosis by interfering with the proteins in the apoptotic pathways (Guo et al., 2009; Wang et al., 2015). Cancer cells exploit the’ cytoprotective function of Hsp70 to sustain themselves (Giri et al., 2017). In HCC and lung cancer, it was observed that both Hsp90 and Hsp70 expression levels were upregulated, leading to proliferation and metastasis of cancerous cells (Leng et al., 2012; Cedrés et al., 2018; Sun et al., 2018; Jiang and Shen, 2020). Cell survival was facilitated through cytochrome c inhibition, regulation of extracellular signal-regulated kinase (ERK), phosphorylation of protein kinase B (Akt) and degradation of apoptotic components (Guo et al., 2009; Wang et al., 2015). Therefore, the upregulated expression levels of Hsp27, Hsp70 and Hsp90, increase the invasion and metastasis of some cancerous cells (Katsogiannou et al., 2014; Zhou et al., 2015; Saha and Anirvan, 2020; Wan et al., 2020). Furthermore, the Hsp70 and Hsp60 proteins present on the surface of cancer cells are implicated in immunomodulation, as they bind and activate immune cells and antibodies (Burgio et al., 2021). Thus, this could explain the possible link between cancer and COVID-19 susceptibility. This association is thought to be due to the dysregulated immune system in both cancer and COVID-19 patients (Zong et al., 2021).

## 5 Cellular stress drives heat shock protein expression

**FIGURE 4:**
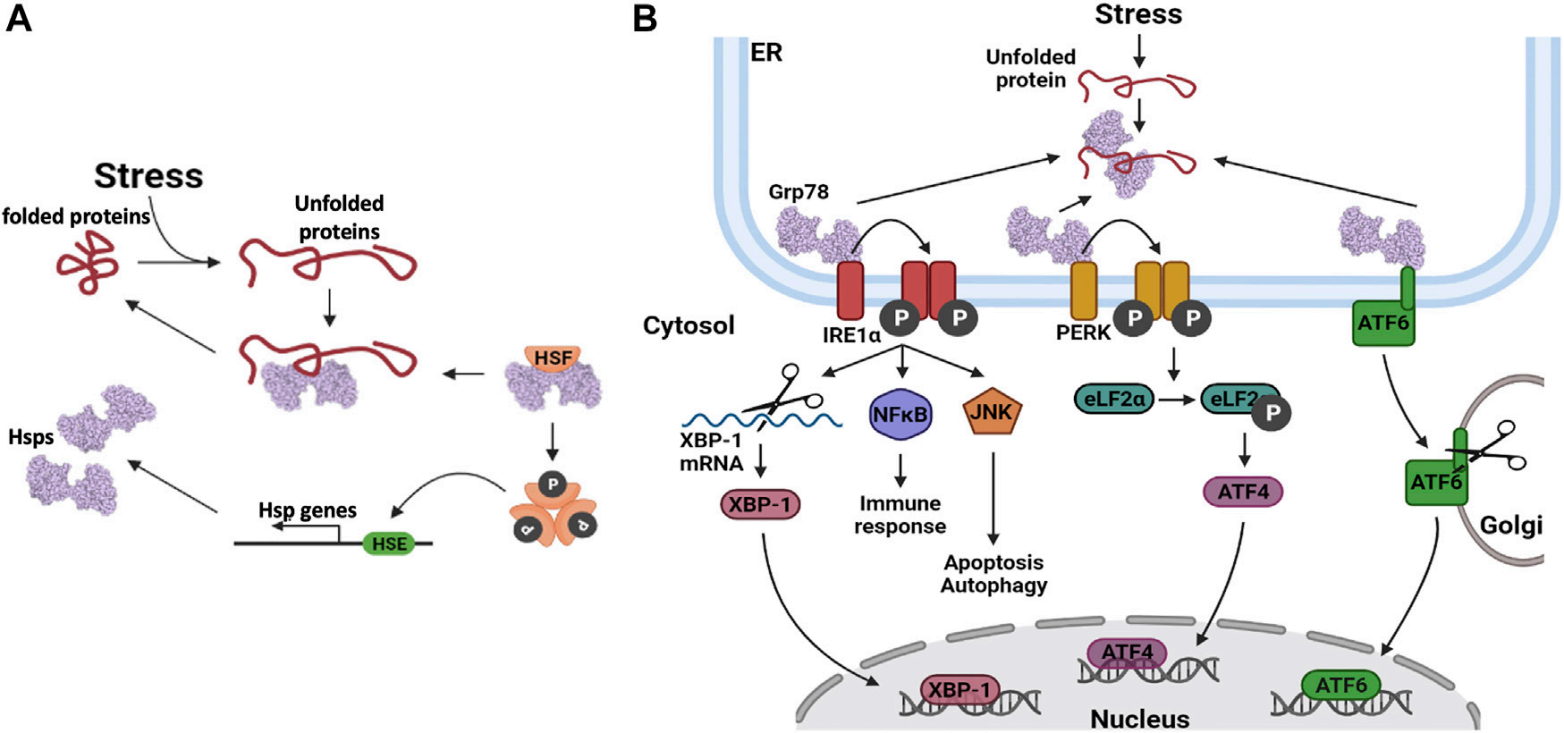
Heat shock protein expression regulation. **(A)** Upregulation of Hsp gene transcription. When unfolded proteins accumulate in the cell, Hsps dissociate from their bound heat shock factors (HSF). This frees up the Hsps to assist in protein folding and it also activates the HSF. The HSFs are phosphorylated, after which they oligomerize. The HSFs bind to heat shock elements (HSE) in the promoter regions of Hsp genes, thereby enhancing their transcription. **(B)** Hsp gene transcription is upregulated via three distinct pathways involving three distinct transcription factors (TFs); Inositol requiring enzyme 1α (IRE1α), protein kinase RNA-like endoplasmic reticulum kinase (PERK) protein and activating transcription factor 6 (ATF6).

Cells that are exposed to stress respond by upregulating some of their Hsp stress proteins (Samali and Orrenius, 1998; Richter et al., 2010). The levels of upregulation are dependent on the type of stress, as the first response for cells is to facilitate post-translational modification (PTM). PTMs on Hsps modulate the chaperone efficiency and enable it to deal with increased demand due to increased protein unfolding. For example, Hsp90 phosphorylation and Hsp70 acetylation have been shown to increase their chaperone activities (Xu et al., 2012; Park et al., 2017). Continued stress stimuli activate the HSR, which is mainly modulated by HSF1 (Sarkar and Roy, 2017). In its inactive state, the HSF is bound to an HSP (Figure 4A). When an HSF is activated, it dissociates from the Hsp and undergoes phosphorylation and oligomerization in the cytosol, after which it translocates into the nucleus (Xu, 2012). The HSF binds to the HSE, located in the promoter regions of HSP genes, which in turn activates the increased expression of the Hsps (Kmiecik and Mayer, 2022).

The ER is also an important organelle in cell stress response as it houses a protein folding machinery. In the wake of external stress sources, the ER-resident Hsps are known to transverse and dissociate from their substrates to become available for the suppression of protein aggregation. Grp78 and Grp94 are central players in the ER protein refolding machinery, and they often cooperate to achieve their chaperone goals (Zhu and Lee, 2015). However, if these proteins are overwhelmed, the unfolded protein response (UPR) is activated. During this event, Hsp expression is upregulated, and cytosolic protein translation is halted to limit the peptide load in the ER (Marzec et al., 2012; Hetz and Papa, 2018). Terminally misfolded proteins that cannot be rescued are channelled to the ERAD pathway for degradation (Oikonomou and Hendershot, 2020; Ninagawa et al., 2021). It should be noted that the PDIs and their associated systems are also responsive to stress as they control proper disulphide bond formation and can also catalyse reverse reactions. In addition, they also distinguish properly and erroneously glycosylated proteins, towards channelling the latter to the ERAD for degradation.

The UPR stress response is activated and regulated via three distinct pathways which enable the expression of UPR related genes (Figure 4B). These three pathways are initiated by the dissociation of Grp78 from the client protein, in response to elevated levels of unfolded proteins in the ER lumen (Lukas et al., 2019). In the Inositol requiring enzyme 1α (IRE1α) pathway, IRE1α undergoes autophosphorylation, followed by oligomerization which facilitates its association with downstream signalling molecules (Hetz et al., 2006; Sano and Reed 2013). Thus, IRE1α interacts with c-Jun N-terminal kinase (JNK) to activate autophagy and with the nuclear factor NFĸB, to activate inflammatory signalling pathways that regulate the release of cytokines and chemokines (Sano and Reed, 2013). IRE1α also has endoribonuclease activity, which is utilized to alternatively splice the X-box binding protein 1 (XBP-1) mRNA, yielding the XBP-1 transcription factor. XBP-1 translocates to the cell nucleus where it activates the transcription of Grp78 and other proteins that are inherent to the ERAD pathway (Kitamura, 2008). The second pathway involves the protein kinase, RNA-like endoplasmic reticulum kinase (PERK). When PERK is released from Grp78, autophosphorylation of PERK is triggered, which in turn phosphorylates the translation elongation initiation factor 2α (eLF2α) (Schönthal, 2012). The subsequent inactivation of eLF2α, relieves ER stress by preventing additional protein synthesis and thereby decreasing the number of proteins that require folding. Transcription factor 4 (ATF4) is translocated to the nucleus, where it activates the expression of numerous stress regulating and pro-apoptotic genes (Lukas et al., 2019). The third pathway involves activating transcription factor 6 (ATF6). Upon dissociation from Grp78, this protein is transported to the Golgi apparatus, where it is proteolytically cleaved. The mature ATF6 protein enters the nucleus where it activates the expression of various UPR and ERAD pathway proteins (Sano and Reed, 2013; Lukas et al., 2019). These events are implicated in the viral life cycle as the coronavirus replication cycle induces ER stress (Sureda et al., 2020). As such, the patient’s UPR/ER stress response may be a predictor of the SARS-CoV2 antiviral response.

## 6 Heat shock protein upregulation could facilitate both viral cellular uptake and replication

Viral proteins, like human proteins, require host chaperones for the folding and assembly of complex viral core particles (Xiao et al., 2010). The upregulation of these host molecular chaperones thus facilitates viral replication (Table 2). Hsps are important in the replication of virtually all viruses including, DNA viruses, both positive and negative sense RNA genomes and double-stranded RNA viruses (Song et al., 2010; Wan et al., 2020). Due to limited data on the role of Hsps on SARS-CoV2 infection, we highlight some of the important pathways from other unrelated viruses that may use unique protein folding systems to draw similarities in COVID-19 pathogenesis. Generally, viral protein homeostasis presents a distinct set of clients for cellular protein folding machinery. As such, viral replication is subject to the folding capacity of the host cell due to three main factors. Firstly, the limited genomes of viruses entail that the viral proteins are multifunctional, and as such, require structurally complex proteins that are solely dependent on chaperones for folding (McBride et al., 2014). Secondly, many cytopathic viruses produce copious amounts of viral proteins within a short time (Oualikene et al., 2000), which places a huge protein folding burden on the host cell. Thirdly, viral capsid precursors are more prone to aggregation and misfolding due to their complexity as they are made of at least a thousand identical subunits (Rossmann, 1984; Geller et al., 2012; Cheng and Brooks, 2013). Several RNA viruses replicate with minimal proofreading and produce several mutant viral proteins during infection, which require a robust host chaperone system to fold into functional forms. In general, CoV manipulate the host chaperone system to render the cells more conducive for their replication. For example, the SARS CoVs E-protein has been implicated in suppressing the stress response in host cells upon infection (DeDiego et al., 2007). It has also been reported that SARS-CoVs structural proteins S, 6, 3a and 8a, induce ER stress response (Ye et al., 2008; Fung et al., 2014; Shi et al., 2019).

**TABLE 2:** The functions of Hsps in RNA virus infections.

**Chaperone family**	**Selected members**	**Function in RNA viral infection**	**Related RNA viruses**	**References**
Hsp90	Hsp90α; Hsp90β	Virus entry into host cell	Enterovirus A71, Dengue, Japanese encephalitis virus	Tsou et al. (2013)
				Reyes-del Valle et al. (2005)
				Cabrera-Hernandez et al. (2007)
		Virus replication	Influenza, Paramyxoviruses: vesicular stomatitis virus, Human parainfluenza virus type 2 and 3, Simian Virus 41 or Chikungunya, Hepatitis C virus	Momose et al. (2002)
				Connor et al. (2007)
				Geller et al. (2013)
				Rathore et al. (2014)
				Ujino et al. (2009)
		Virus protein maturation and assembly	Hepatitis C virus, Influenza, Picornaviruses, Poliovirus, Rhinovirus, Coxsackievirus, Noroviruses	Waxman et al. (2001)
				Geller et al. (2007)
				Vashist et al. (2015)
				Kumar et al. (2019)
		Cellular transformation	Human T-lymphotropic virus	Ikebe et al. (2014)
Hsp70	Grp78; Hsc70; Hsp70; Hsp72	Virus entry into host cell	Chicken Anaemia virus-9, Enterovirus A71, Dengue, Japanese encephalitis virus, Zika virus, Human T-lymphotropic virus, human immunodeficiency virus -1	Triantafilou et al., 2002, Xu et al., 2019
				Vega-Almeida et al. (2013)
				Das et al. (2009)
				Taguwa et al. (2019)
				Pujhari et al. (2019)
				Sagara et al. (1998)
				Fang et al. (1999)
				Agostini et al. (2000)
				Reyes-del Valle et al. (2005)
		Virus replication	Mumps virus, Canine distemper virus, Hepatitis C virus, Respiratory syncytial virus, Ebola virus, Influenza, SARS-CoV2	Katoh et al. (2015)
				Vasconcelos et al. (1998)
				Oglesbee et al. (1993)
				Chen et al. (2010)
				Oliveira et al. (2013)
				García-Dorival et al. (2016)
				Nelson et al. (2017)
				Manzoor et al. (2014)
		Virus gene expression	Coxsackievirus B3, Enterovirus A71, Influenza A	Wang et al. (2017)
				Dong et al. (2018)
				Lee et al. (1994)
				Melville et al. (1999)
		Virus assembly	Reovirus, Poliovirus, Coxsackievirus B1, Influenza	Leone et al. (1996)
				Macejak and Sarnow, (1992)
				Hirayama et al. (2004)
		Virus release	Hepatitis C virus	Khachatoorian et al. (2014)
				Khachatoorian et al. (2015)
Hsp60	Hsp60; TRiC; GroEL; Hsp58; yHsp60	Immunomodulation	Japanese encephalitis virus, Influenza, Dengue	Swaroop et al. (2018)
				Graef et al. (2010)
				Fislová et al. (2010)
				Karlas et al. (2010)
				Padwad et al. (2009)
		Apoptosis regulation	Hepatitis C virus, Rotavirus	Kang et al. (2009)
				Chattopadhyay et al. (2017)
		Genome integration	Human immunodeficiency virus	Bartz et al. (1994)
				Parissi et al. (2001)
Hsp40	Hdj2; DnaJB1; DnaJA1; DnaJA1; DnaJC14; DnaJA3; Hdj1; hTid1; DnaJB6; ERdJ5	Virus entry into host cell	Human immunodeficiency virus	Chiang et al. (2014)
				Ko et al. (2019)
		Virus replication	Japanese encephalitis virus, Influenza	Wang et al. (2011)
				Batra et al. (2016)
				Cao et al. (2014)
		Virus gene expression	Influenza, Human immunodeficiency virus	Melville et al. (1997)
				Sharma et al. (2011)
				Saksela, (1997)
				Simmons et al. (2001)
				Doms and Trono, (2000)
		Virus protein maturation	Yellow fever virus	Yi et al. (2012)
		Immunomodulation	Hand foot and mouth disease virus	Zhang et al. (2019)
Small Hsps	Hsp27	Virus replication	Enterovirus A71, Classical swine fever virus	Ling et al. (2018)
				Sun et al. (2017)
PDIs	PDI; ERp57	Virus entry into host cell and uncoating	Dengue, Human immunodeficiency virus	Gallina et al. (2002)
				Bi et al. (2011)
				Diwaker et al. (2015)
		Virus translation	Enterovirus A71	Wang et al. (2016)
		Oxidative stress and ER stress	Influenza, Hepatitis C virus, Encephalomyocarditis virus, Respiratory syncytia virus, Japanese encephalitis virus, Human immunodeficiency virus	Knobil et al. (1998)
				Korenaga et al. (2005)
				Ano et al. (2010)
				Olagnier et al. (2014)
				Liao et al. (2002)
				Dobmeyer et al. (1997)

### 6.1 Viral entry

**FIGURE 5:**
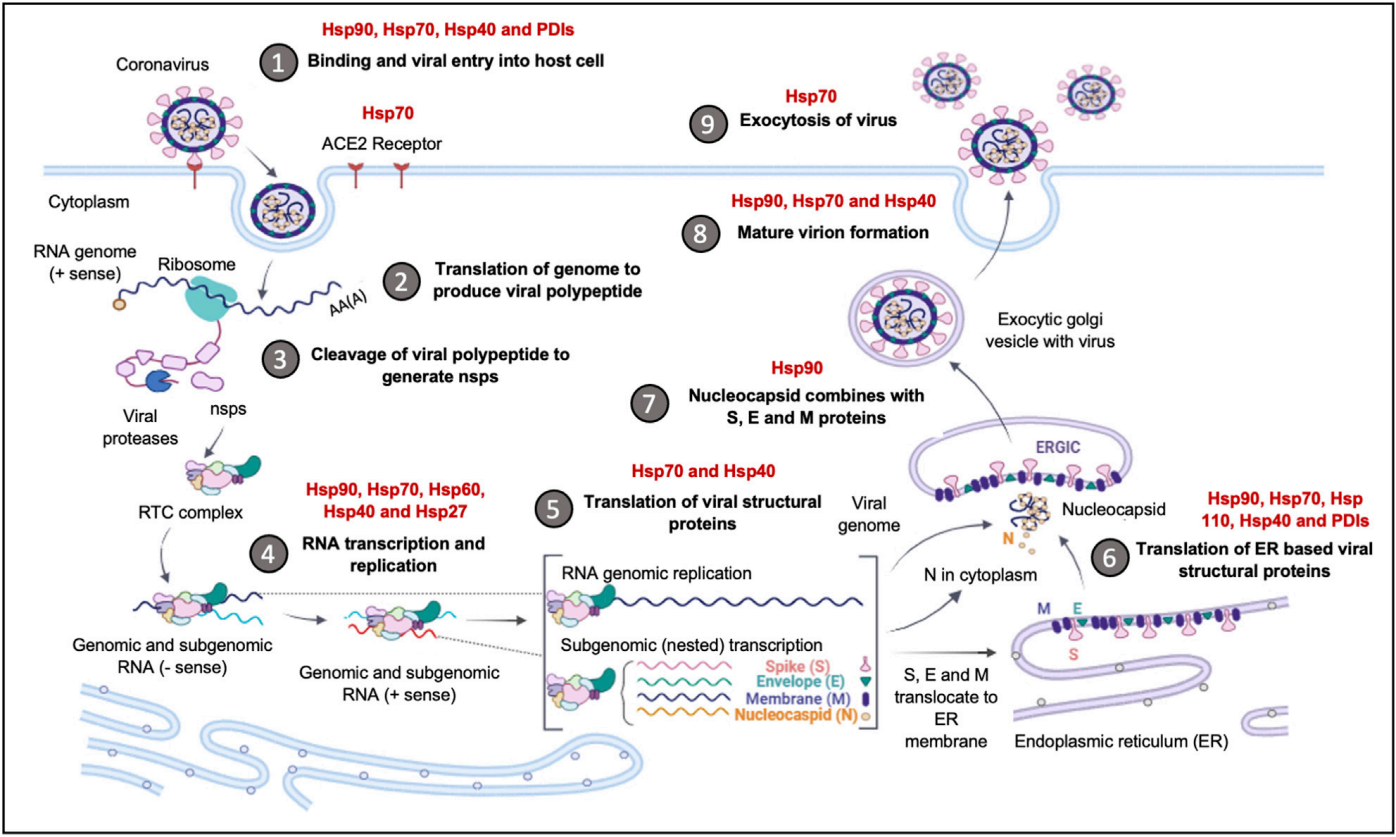
The upregulation of Hsps facilitates Coronavirus invasion and replication. The Hsps implicated in the Coronavirus replication cycle are highlighted. The binding and entry of the viral particle is achieved through the ACE-2 receptor, with Hsp90, Hsp70, Hsp40 and PDI, acting as auxiliary receptors. Upon viral entry and initial translation and transcription, Hsp40, Hsp70, Hsp60 and Hsp27, are recruited to facilitate in viral protein maturation. The folding of structural proteins is assisted by Hsp70 and Hsp90. The ER-resident chaperones, Grp94, Grp78, ERDnaJ and PDIs, facilitate the folding and insertion of viral proteins onto the ERGIC membrane. The formation of the nucleocapsid complex is overseen by Hsp90, while viral exocytosis requires the cooperation of Hsp90, Hsp70 and Hsp40. Image created with Biorender.

Several viruses interact with Hsps as auxiliary receptors to enter host cells through the clathrin-mediated endocytosis pathway. Hsp70 is commonly implicated as an auxiliary receptor (Figure 5), as is observed in the host cell entry of zika virus; a process that is facilitated by extracellular Hsp70 (Pujhari et al., 2019). The ER Hsp70 homology, Grp78, has been reported to facilitate the invasion of host cells by several viruses, amongst them, the Japanese encephalitis virus (Nain et al., 2017) and SARS-CoV2 (Ha et al., 2020; Carlos et al., 2021). The SBD of Grp78 positioned on the surface of African green monkey kidney epithelial Vero cells, was reported to recognize the S protein RBD of the SARS CoV2 to facilitate viral entry into these cells. In addition, the upregulated expression of Grp78 was associated with the surface expression of the ACE2 receptors in SARS-CoV2 patients (Sabirli et al., 2021). The interaction of the ACE2 receptors with Grp78 required both the NBD and SBD (Carlos et al., 2021). These findings suggest that the full-length human Grp78 protein and possibly its functional partners Grp94, Hsp40 and PDIs could be targeted to reduce SARS-CoV2 entry into host cells and to combat the ensuing viral infection.

### 6.2 Virus replication

The central role of Hsp90 in protein complex formation is thought to function as a catalyst for the replication of invading viruses (Wan et al., 2020). Hsp90 acts as a buffer, preventing deleterious folding outcomes of mutated viral proteins (Blagosklonny et al., 1996; Jaeger and Whitesell, 2019). As such, its role in facilitating fold of mutation prone viral proteins is apparent. Both Hsp40 and Hsp70 are virtually involved in virtually all the stages of viral infection in the host cell, as they modulate viral entry, replication, gene expression, and virion assembly and release (Table 2). In addition, the Hsp40 protein, DnaJB6 is also involved in immunomodulation of the foot-and-mouth-disease virus (FDV) infection (Zhang et al., 2019), and it is therefore plausible that these Hsps play a role in viral replication.

### 6.3 Viral protein maturation and viral assembly

Hsp70/Hsp90 proteins are the main drivers of protein folding within host cells and, inadvertently, they also facilitate the folding of invader viral proteins, enabling them to attain functional states. Protein folding occurs mainly in the cytosol of the infected cell and the Hsp40 chaperones are involved in the recruitment of the viral proteins from the ribosomes. Several of the replication steps of the Coronavirus, such as translation, takes place on the ER membranes (Knoops et al., 2008). Some of the viral proteins are thus, translocated across the ER membrane during translation, while others remain in the cytosol for folding, after which they are recruited to the ER for virion assembly. A distinct set of chaperones control the folding processes of the proteins within the ER lumen. The SARS-CoV2 structural proteins S, 6, 3a and 8a, require folding inside the ER lumen. The increased load of viral proteins in the ER disrupts the ER protein folding machinery, which induces ER stress (Fung et al., 2014; Shi et al., 2019). SARS-COV2 viruses are so dependent on the integrity of the ER folding machinery for their own replication, the virus inhibits the ER stress cascade mechanisms by the activation of the XBP-1 mediated pathway of the UPR, thereby preventing apoptosis. This is potentially mediated by the E protein; a 76 amino acid protein that oligomerises to form an ion conductive pore in the ER membranes, which restores the Ca^2+^ imbalance generated during ER stress (Torres et al., 2006). Viral proteins heavily depend on Hsp90 for their folding, assembly, and maturation, which partly accounts for the induction of Hsp90 expression (Ha et al., 2020).

## 7 Role of heat shock proteins in inflammation

SARS-CoVs cause ARDS (Reizine et al., 2021), hemophagocytic syndrome (Yang et al., 2021), lymphoid depletion (Silverstein et al., 2022) and skeletal muscle fibre necrosis (Suh et al., 2021), which are all consequences of elevated levels of pro-inflammatory cytokines. Although predominantly expressed and found intracellularly, Hsp90 and Hsp70 were reportedly found in the extracellular space surrounding both stressed and non-stressed cells (Tsutsumi and Neckers, 2007). Surface exposed Hsp90 is thought to be involved in immunomodulation and facilitates the internalisation of viruses such as dengue virus (DENV), infectious bursal disease virus (IBDV) and influenza A virus (Reyes-del Valle et al., 2005; Cabrera-Hernandez et al., 2007; Wang X. et al., 2020). In line with recent evidence, SARS-CoV2 has been linked to the stimulation of stress proteins (Gadotti et al., 2021) which may potentially facilitate virus internalisation and immunomodulation. SARS-CoV2 infection potentially utilises molecular mimicry to imitate host cell surface receptors facilitating entry and evading the host immune response (Angileri et al., 2020; Cappello, 2020; Cappello et al., 2020; Hightower and Santoro, 2020; Kasperkiewicz, 2021). The molecular mimicry of human cell surface-expressed molecules could result in the expression of antibodies that cross-react with human proteins. This culminates in an autoimmune response from the host (Burgio et al., 2021). Hsps are some of the molecules that share epitopes with microbial counterparts and are often the targets for molecular mimicry (Burgio et al., 2021). It was reported that some Hsps share both immunogenic and antigenic epitopes with SARS-CoV2 viral proteins (Marino Gammazza et al., 2020). This partly explains the cause of an increase in autoimmunity cases in SARS-CoV2 patients.

The cytokine storm that results from a SARS-CoV2 infection is the major cause of lung damage resulting in mortality (Song et al., 2020). A cytokine storm refers to the excessive production of proinflammatory cytokines such as interleukins 1 and 6 (IL-1 and IL-6), tumour necrosis factor α (TNF-α) and interferon -y (Huang et al., 2005; Copaescu et al., 2020). The release of these cytokines triggers a flood of immune cells to the infection site, including T cells, macrophages, and neutrophils, among others. This excessive immune response results in tissue and organ damage, which may ultimately lead to organ failure and, by extension, untimely death (Ragab et al., 2020).

Hsps facilitate the presentation of antigens by the major histocompatibility complex (MHC-1) on the surface of coronavirus infected cells, for clearance by the NK and CD8+T cell subsets (Moseley, 2000; Furuta and Eguchi, 2020; Neukirch et al., 2020). Hsp90 was shown to not only function as chaperone, but also as an antigen-presenting molecule during lymphocytic choriomeningitis viral infection (Basta et al., 2005). In addition, Hsp90 immunomodulates the host response to Sendai virus infection by regulating the activation of interferon regulatory factor 3 and TBK-1 stabilization of Sendai virus (Yang et al., 2009). Generally, Hsps are upregulated to assist in the assembly and folding of numerous immune system antigen-recognition proteins. These include molecules such as immunoglobulins, T-cell receptors and components of the MHC (Zugel and Kaufmann, 1999). Furthermore, the ER-resident Grp94, BiP and calnexin, are involved in the assembly of antibody light and heavy chains in the ER lumen (Zugel and Kaufmann, 1999). It has also been suggested that Hsps may take part in antigen delivery to MHC proteins, due to their promiscuity in binding a wide range of similar peptides (Zininga et al., 2018). These diverse roles Hsps make them important immune modulators that promote viral infection.

## 8 Conclusion and future perspectives

Hsp90 plays a central role in cellular protein quality control. Inhibition of the Hsp90 system affects various signalling pathways, making it an attractive drug target in viral infections. Additionally, the high mutation rates of RNA viruses further support targeting Hsp90 as an antiviral candidate. In many viral infections, Hsp90 was shown to be an important chaperone that functions in facilitating viral protein folding, replication, transport, and assembly (Geller et al., 2013; Geller et al., 2018; Wyler et al., 2021). Most Hsp90 inhibitors were initially identified as anticancer agents and some of these are being repurposed for use against viral infections (Ramos and Ayinde, 2021; Zhao et al., 2022). Hsp90 inhibitors are potential broad action antivirals, as they act on several viruses by blocking their replication and stimulating apoptosis in infected host cells. It was recently reported that Hsp90 inhibitors prevented endothelial barrier damage in lung tissues and reduced SARS-CoV2 replication (Kubra et al., 2020; Lubkowska et al., 2021; Wyler et al., 2021). Another study hypothesised that Hsp70 and Hsp90 proteins potentially bind to the ACE2 receptors, masking the receptors and inhibiting binding to SARS-CoV2 viruses, resulting in reduced viral host cell entry (Alreshidi et al., 2021). These Hsp90 inhibitors exhibiting potent antiviral activity were reportedly effective at lower concentrations compared to the dosages required in cancer treatments. Therefore, there is merit in targeting the Hsp system, as the shorter acute nature of viral infection implies the use of much less total dosage compared to that required to treat cancer. Taken together, the use of Hsp inhibitors offers promising prospects as combinational treatments in controlling COVID-19.
